# Quantitative profiling of basal and stress-induced ribosome collisions

**DOI:** 10.64898/2026.07.21.739814

**Published:** 2026-10-06

**Authors:** James Marks, Pedro H Ayres-Galhardo, Bavavarshini Tamilselvam, Alexis B Roberson, Ruby Pitts, Nicholas R Guydosh, Sezen Meydan

## Abstract

**Background:**

Ribosomes stall when they encounter problematic codons or cellular stress that perturbs translation. Stalled ribosomes can lead to the formation of ribosome collisions, also known as disomes, that engage cellular surveillance and stress signaling pathways. How many disomes form during basal conditions and how disome levels change under stress remain poorly understood.

**Results:**

We used spike-in normalized Ribo-seq and Disome-seq to quantify transcriptome-wide disome levels. Applying this approach in yeast and human cells, we found that disomes generate approximately 2-10% of all footprints under basal conditions. A high-resolution Disome-seq experiment in human cells identified reproducible disome-forming sites that contribute to the basal level of disome formation in the cell. Exposure of yeast cells to methyl methanesulfonate and human cells to anisomycin increased disome abundance up to four-fold and redistributed collisions in a stress-specific and context-dependent manner.

**Conclusions:**

These data provide a quantitative, transcriptome-wide framework for measuring disome levels and reveal how translational stress reshapes the landscape of ribosome collisions in cells.

## Full Text Availability

The license terms selected by the author(s) for this preprint version do not permit archiving in PMC. The full text is available from the preprint server.

