# Supplementary material for: Quantitative profiling of basal and stress-induced ribosome collisions
Source: bioRxiv. 2026 Jul 22:2026.07.21.739814. Preprint. [Version 1] doi: 10.64898/2026.07.21.739814 (PMC13419767; doi:10.64898/2026.07.21.739814)

bioRxiv preprint doi: https://doi.org/10.64898/2026.07.21.739814; this version posted July 22, 2026. The copyright holder for this preprint (which was not certified by peer review) is the author/funder. This article is a US Government work. It is not subject to copyright under 17 USC 105 and is also made available for use under a CC0 license.

SUPPLEMENTAL MATERIAL

Figure S1: Sequencing depth affects identification of strongly enriched disome sites. Related to Figure 2.

(A) Computational pipeline used to map downsampled footprint reads. The reads were adapter-trimmed and demultiplexed. The reads were downsampled to a defined number of reads and were subsequently mapped to non-coding RNAs. Unmapped reads were deduplicated and mapped to transcriptome. Disome peak score analysis was conducted as described in Methods.
(B) Left: pLogo analysis showing the consensus amino acid sequence of the disome sites with a disome peak score >2 (left) or >10 (right) after downsampling the reads to $10x10^6$ (top panel) or $1x10^6$ (bottom panel). Overrepresented and underrepresented sequences are shown at each plogo image, where the significance cutoff (p<0.05) is indicated with a red line. Right: Disome-seq snapshot of *XBP1-unspliced* transcript after downsampling the reads to $10x10^6$ (top panel) or $1x10^6$ (bottom panel).

Figure S2: MMS treatment affects the distribution of ribosomes across open reading frames. Related to Figure 3.
(A) Replicate 2 of western blotting of yeast cells treated with 0.1% MMS for 30 minutes.
(B) Metagene analysis of Ribo-seq (top) and Disome-seq (bottom) average normalized reads corresponding to region surrounding the start (left) and stop (right) codons in mock- and MMS-treated cells. The shaded error bar indicates mean ± standard deviation from 2 biological replicates. Y axes indicate the distance of the start or stop codon from the 3' end of the footprints.

Table S1. Sequences of oligonucleotides used in this study**.**

Table S2: Read numbers and disome percentage values from different pipelines.

Table S3. Outputs from disome peak score to support Figure 2.

bioRxiv preprint doi: https://doi.org/10.64898/2026.07.21.739814; this version posted July 22, 2026. The copyright holder for this preprint (which was not certified by peer review) is the author/funder. This article is a US Government work. It is not subject to copyright under 17 USC 105 and is also made available for use under a CC0 license.

Figure S1

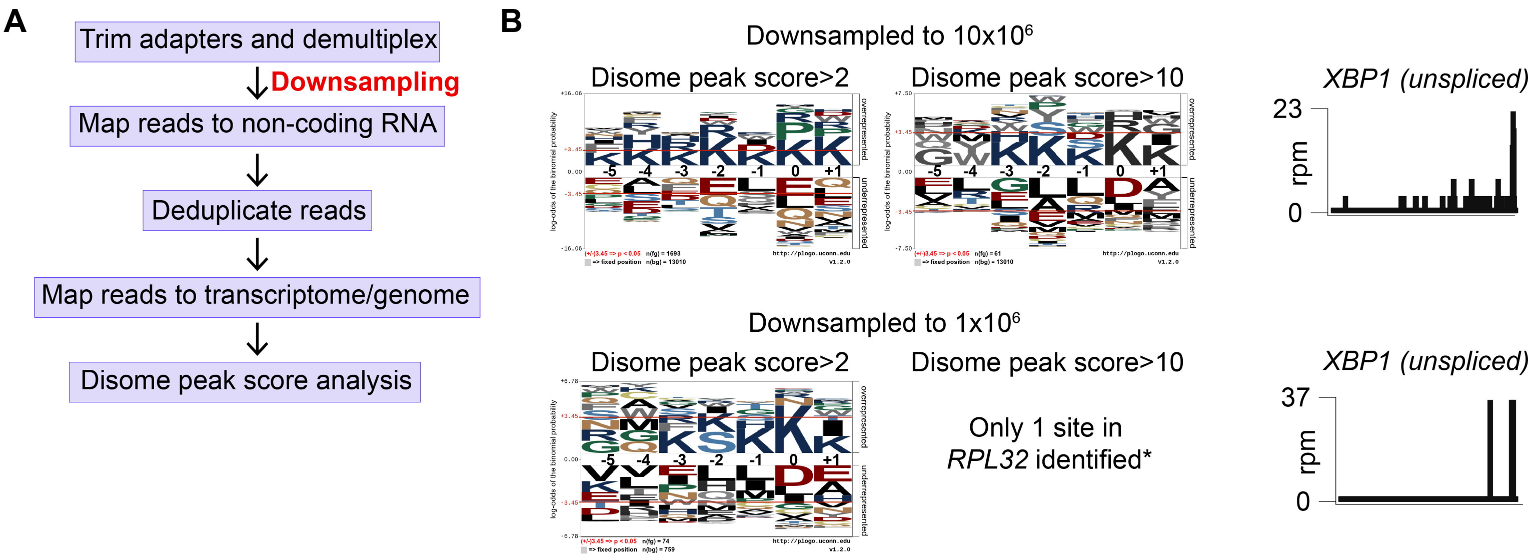

bioRxiv preprint doi: https://doi.org/10.64898/2026.07.21.739814; this version posted July 22, 2026. The copyright holder for this preprint (which was not certified by peer review) is the author/funder. This article is a US Government work. It is not subject to copyright under 17 USC 105 and is also made available for use under a CC0 license.

Figure S2

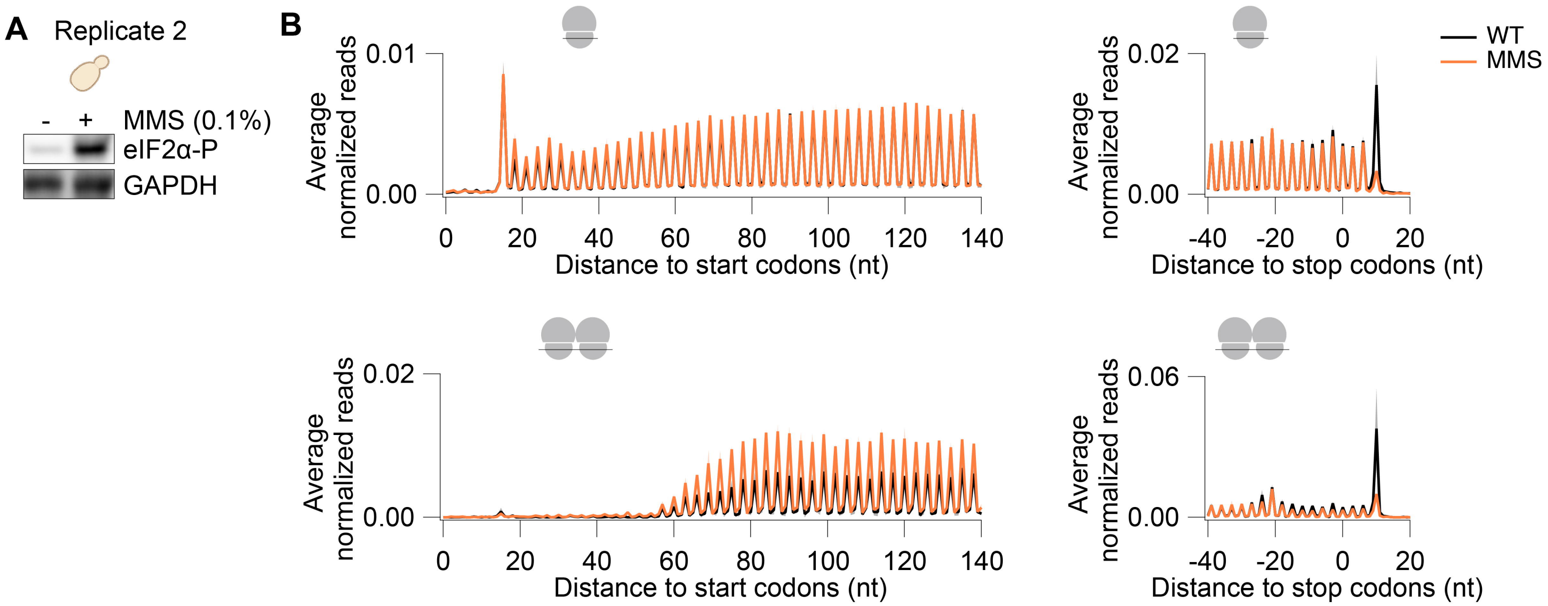

Supplement: 1 [file NIHPP2026.07.21.739814v1-supplement-1.pdf]
